# Quality of Life and Its Biopsychosocial Determinants: A Study Among the Yadav Community From Delhi, India

**DOI:** 10.7759/cureus.54690

**Published:** 2024-02-22

**Authors:** Kirti Rao, Vaidehi Goswami, Shivani Chandel

**Affiliations:** 1 Anthropology, University of Delhi, Delhi, IND

**Keywords:** mental health, anxiety, depression, hypertension, obesity, quality of life (qol)

## Abstract

Introduction

The COVID-19 pandemic has not only affected the physical and mental health of people but has also had a detrimental impact on their quality of life (QoL). Therefore, ways to improve the QoL must be promoted for the overall well-being of individuals and society. The present study aims to assess the status of QoL and understand its association with physical and mental variables among the Yadav community of Delhi.

Methods

A cross-sectional study was conducted among 600 participants aged 18 to 55 years. Participants were recruited based on inclusion criteria, that is, individuals aged between 18 and 55 years, residing in Delhi, belonging to the Yadav community, and exclusion criteria, that is, pregnant females, lactating mothers, and individuals with any chronic illness or suffering from COVID-19. Data were analyzed in IBM SPSS Statistics for Windows, Version 22 (Released 2013; IBM Corp., Armonk, New York) using various descriptive and inferential statistics.

Results

Mental disorders were found to have a negative impact on QoL. The participants detected with higher levels of stress and depression reported a significant decrease in their scores (p ≤ 0.001) across all the domains of QoL. Hypertensive individuals have significantly lower mean scores than normal individuals across all domains. The regression analysis revealed that all these predictors have a negative impact on QoL. The present study indicated that women have a lower QoL than men. Among the four domains of QoL, the participants in the social domain had the highest proportion of good QoL, followed by the environmental domain.

Conclusion

This study reveals that the predictors of physical and mental health adversities have a negative association with QoL, and the results were significant across all the domains. It affects an individual's overall well-being, leading to decreased productivity, work-life balance, and happiness. The status of QoL among the participants was poor in the psychological domain and good in the social domain. Intervention programs based on diverse sociocultural practices should be targeted toward improving QoL by understanding the health needs and risks of different communities in Delhi.

## Introduction

Quality of life (QoL) is a multifarious and comprehensive concept that includes various dimensions influenced by an individual's perception. It is defined as an individual's subjective evaluation of their position in life, taking into account the cultural and value systems in which they live concerning their goals, concerns, expectations, and standards [1]. QoL is essential in assessing people's health. It commonly focuses on an individual's physical and mental health and functional performance. However, it can be measured in a broad range. The domains of QoL can be affected by age, sex, rural and urban areas, culture, marital status, education, employment, socioeconomic status, health, and disease status [2].

The COVID-19 pandemic has significantly changed social interactions, work, education, and leisure activities. The outbreak of this infectious disease negatively affected the physical, social, and psychological functioning of individuals and society. This disease impacted not only the physical health of individuals, resulting in many fatalities worldwide but also their QoL [3].

Mental disorders are among the leading causes of nonfatal disease burden in India and have been identified as one of the most critical factors in QoL [4]. Research on the viral outbreak has broadly indicated adverse outcomes such as depression and anxiety, which negatively influence QoL [5].

One of the biggest public health challenges worldwide is the silent pandemic of chronic diseases, which includes obesity and hypertension, gradually spreading to all countries [6]. QoL is an essential indicator for evaluating hypertensive individuals. Hypertension is one of the most crucial risk factors for cardiovascular disease leading to mortality. A systematic review found that hypertensive patients had a lower QoL compared with normotensive individuals [7]. On the other hand, obesity alone can impact QoL as much as any other chronic medical condition. Literature suggests that when it becomes comorbid with other diseases, the impact of this illness is magnified [8].

Sedentary lifestyles can also significantly impact an individual's QoL. It is a well-known fact that people in urban areas, including Delhi, are leading sedentary lifestyles due to various reasons such as long commutes, desk-bound jobs, lack of physical activity, and easy access to unhealthy food options. This has led to an increase in lifestyle diseases such as obesity, hypertension, and diabetes, which have a direct impact on the QoL of individuals [9]. Delhi, the capital of India, is a bustling metropolitan city with a fast-changing pace of life. Therefore, it is crucial to study the QoL of people in Delhi, as it can have a significant impact on the health of individuals and communities in the city.

Moreover, the assessment of the QoL of individuals provides a comprehensive framework for addressing various aspects of human well-being. Dimensions captured through QoL scales will directly or indirectly help in achieving sustainable development goals and improve India’s rank in the World Happiness Report. The parameters on which the happiness index is measured are quite similar to those of QoL; both are evidently not met [10]. The attainment of QoL is affected and disrupted in today's world, which is riddled after the COVID-19 outbreak. This has become a matter of concern, and more research on this should be promoted. Therefore, the present study aims to assess the status of QoL and its association with sociodemographic factors, physical health (obesity and hypertension), and psychological health (stress, anxiety, and depression) variables among the Yadav community residing in Delhi, India, to know how these factors impact their QoL.

## Materials and methods

Study participants

A cross-sectional study was conducted among the Yadav community of Delhi. A total of 600 participants (males: 259, and females: 341) aged 18 to 55 years were included in the study. All the participants were recruited randomly using the household survey technique. The sample size for the proposed study was calculated using Daniel’s formula. The prevalence of obesity among adults in Delhi was 38% [11]. The formula used [12] is: n = (Z^2^ P(1 - P))/d^2^, where n is the sample size, Z is the Z statistic for a level of confidence, P is the expected prevalence, and d is the precision. Z for a 95% level of confidence is 1.96. The precision is 4% or 0.04. P, the prevalence found in the literature, is 0.38.

They refer to peasant-pastoral communities or castes in India, an endogamous population, one of the oldest communities residing in Delhi. This caste group also belongs to the Other Backward Classes of India. The Yadavs mostly live in the northern parts of India. Primarily involved in cultivation, occupied in raising cattle or the milk business, their traditional occupations have changed over time, and economic advancement has progressed through involvement in cattle-related businesses to transportation and construction [13].

Participants were recruited based on inclusion and exclusion criteria. The inclusion criteria included (1) individuals aged between 18 and 55 years; (2) individuals belonging to the Yadav community; (3) individuals residing in Delhi; and (4) those who provided written informed consent. The exclusion criteria included those individuals aged less than 18 years and more than 55 years, pregnant females, lactating mothers, and individuals with any chronic illness or suffering from COVID-19, clinically diagnosed with any mental health conditions. Ethical approval was granted by the Departmental Ethical Committee, Department of Anthropology, University of Delhi, India (Ref. No. Anth/2022-23/868). The study was performed in line with the principles of the Declaration of Helsinki.

Data collection

Sociodemographic information comprising age, sex, education, occupation, marital status, family type, number of family members, socioeconomic status, and lifestyle habits was collected from participants using pretested and modified interview schedules. Face-to-face interviews were conducted with the participants to collect this information. The Kuppuswamy Scale 2021 was used to calculate the socioeconomic status of the community [14].

Tools

Quality of Life

The World Health Organization Quality of Life (WHOQOL-BREF) instrument, cross-culturally relevant, was used to evaluate the QoL [15]. The questionnaire comprises 26 self-administered items and is available in 19 languages. The first two items independently examine overall perceptions of health-related QoL (HRQOL), while the following 24 questions assess the four major HRQOL domains defined by the World Health Organization (WHO): physical health, psychological health, social relationships, and environment. The tool uses a scoring method in which the values from all four domains are added together and scaled in a positive direction. The raw scores were calculated for each domain and then transformed to a range between 0 and 100, with higher scores indicative of good QoL. However, it was decided to classify participants into those with scores less than the mean (poor QoL) and those with scores equal to or more than the mean (good QoL).

Stress

This research utilized the 10-item Perceived Stress Scale (PSS-10) inventory to report an individual's experience of stress. The tool measures the psychological manifestations of stress, with scores ranging 0-13 indicating low stress, 14-26 moderate stress, and 27-40 high stress [16].

Anxiety

The Beck Anxiety Inventory (BAI) is a self-reporting tool that assesses the severity of generalized anxiety symptoms, focusing on somatic symptoms of anxiety. The tool has 21 items, with scores ranging from 0-21 indicating low anxiety, 22-35 moderate anxiety, and 36 or higher high anxiety [17].

Depression

The Beck Depression Inventory - Second Edition (BDI-II) is among the most widely used instruments for screening the presence and severity of depressive disorder symptoms in clinical and nonclinical settings [18]. The BDI-II comprises 21 items answered on a 4-point scale regarding the occurrence of depressive symptomatology in the previous two weeks. It is suitable for community screening and studies with large samples, given that this scale is relatively short, self-administered, and has an easy scoring procedure.

Blood Pressure Assessment

Blood pressure was measured following a standardized protocol. Prior to measuring, it was ensured that the participant had not eaten anything or taken tea or coffee in the last half an hour. Participants were asked to rest quietly in a seated position for 30 minutes before the measurements were taken. A digital sphygmomanometer (HEM-7156, OMRON Co. Ltd., Vietnam) was used for measuring blood pressure. Hypertension status was defined according to the American College of Cardiology/American Heart Association (ACC/AHA) hypertension treatment guidelines [19].

Somatometric Measurements

Somatometric measurements (height in centimeters and body weight in kilograms) were taken following standardized procedures as per the International Society for the Advancement of Kinanthropometry (ISAK) guidelines. Height was measured using an anthropometric rod (Galaxy, India), and body weight was measured using a portable weighing scale (Omron Krups, India). These measurements were used to compute BMI as body weight in kilograms divided by the square of height in meters (kg/m^2^). BMI status was categorized according to the WHO Asian criteria [20].

Statistical Analysis

Histograms and descriptive statistics were used to summarize the studied population's background characteristics and the prevalence of QoL. Continuous variables were compared across categories using analysis of variance (ANOVA) and t-test. Pearson correlation was used to find the correlation between predictors and QoL. Multiple linear regression analysis was performed with age, gender, employment status, socioeconomic status, smoking status, alcohol consumption, BMI, systolic blood pressure, diastolic blood pressure, stress, anxiety, and depression as independent variables with QoL and its four domains (physical, psychological, social, environmental, and total score of QoL) as dependent variables; tests were run separately to ascertain the effect of various predictors on QoL. A p-value less than 0.05 was considered statistically significant for the present study. Data were analyzed using IBM SPSS Statistics for Windows, Version 22 (Released 2013; IBM Corp., Armonk, New York).

## Results

A total of 600 participants from the Yadav community of Delhi, India, participated in the study. They belonged to the age group of 18 to 55 years. Table 1 presents the prevalence of good and bad QoL based on their mean score across the four domains of QoL. It depicts that the parameters for good QoL were highest for the social domain (77.6%, n=466), followed by the environmental domain (58.6%, n=352), the physical domain (55.5%, n=333), and lowest for the psychological domain (50.8%, n=305).

**Table 1:** Overall prevalence of quality of life among the studied population

Variables	Categories	Overall n (%)
Physical	Good	333 (55.5)
	Bad	267 (45.5)
Social	Good	466 (77.6)
	Bad	134 (22.3)
Psychological	Good	305 (50.8)
	Bad	295 (49.1)
Environmental	Good	352 (58.6)
	Bad	248 (41.3)

Table 2 represents the sociodemographic profile of the studied population. Here, distribution was depicted across age and gender. Females (56.8%, n=341) were slightly higher in number compared to male (43.2%, n=259) participants. As Delhi is a metropolitan city, almost all the participants were literate (99.5%, n=597). Three-fourths of the participants were married, but in terms of employment status, more than half were unemployed (58.3%, n=350). Socioeconomic status revealed that nearly 80.2% (n=481) of participants belonged to the middle socioeconomic status category and 17.2% (n=103) to the lower socioeconomic status. Consumption of alcohol (22.5%, n=135) was more prevalent as compared to smoking (9%, n=54) among the participants.

**Table 2:** Baseline sociodemographic characteristics SES: socioeconomic status

Variable	Frequency	Percentage (%)
Age group (years)		
18-25	118	19.7
26-35	186	31.0
36-45	167	27.8
46-55	129	21.5
Gender		
Male	259	43.2
Female	341	56.8
Literacy		
Literate	597	99.5
Illiterate	03	0.5
Marital status		
Unmarried	142	23.7
Married	433	72.2
Widowed	24	4.0
Divorced	01	0.2
Employment status		
Unemployed	350	58.3
Employed	106	17.7
Self-Employed	144	24.0
SES		
Upper	16	2.7
Middle	481	80.2
Low	103	17.2
Alcohol consumption		
No	465	77.5
Yes	135	22.5
Smoking status		
No	546	91.0
Yes	54	9.0

Table 3 shows the comprehensive analysis of the difference in mean scores of various domains of QoL for the possible predictors. A significant difference and decrease in the mean score of the physical (78.54 ± 9.99 vs. 65.09 ± 15.25) and social (74.10 ± 11.61 vs. 71.07 ± 10.67) domains was observed with an increase in age. Males had significantly higher average scores for all the domains (physical: 76.91 ± 11.92; psychological: 68.54 ± 16.82; social: 74.16 ± 10.76) of QoL except the environment domain. The employed participants (physical 75.78 ± 12.32 vs. 69.42 ± 14.44; psychological 68.06 ± 15.42 vs. 61.27 ± 16.63; social: 74.15 ± 10.53 vs. 70.98 ± 11.24; environment: 70.08 ± 10.68 vs. 68.28 ± 10.63) and those who consume alcohol reported significantly higher scores (physical: 76.95 ± 11.35 vs 70.65 ± 14.31; psychological: 70.99 ± 14.06 vs 62.10 ± 16.59; social: 74.31 ± 9.44 vs 71.72 ± 11.43; environment: 70.94 ± 9.79 vs 68.48 ± 10.87) of QoL across all the domains than unemployed and nonalcoholic individuals.

**Table 3:** Analysis of possible predictors affecting quality of life among the studied population p-value ≤0.001 or ≤0.05 is considered significant. SES: socioeconomic status, BMI: body mass index, SD: standard deviation.

Variables	Domains of quality of life (mean ± SD), n= 600			
	Physical	Psychological	Social	Environment
Age (years)				
18-25	78.54±9.99	66.99±17.46	74.10±11.61	71.18±11.84
26-35	74.54±12.37	64.72±16.39	73.25±11.44	68.94±10.97
36-45	70.15±14.26	62.03±15.59	70.92±10.30	67.99±9.56
46-55	65.09±15.25	64.25±16.53	71.07±10.67	68.54±10.62
P value	≤0.001	0.07	≤0.05	0.08
Gender				
Male	76.91 ±11.92	68.54 ±16.82	74.16± 10.76	69.95 ±10.97
Female	68.54 ±14.26	60.73± 15.38	70.89 ±11.09	68.33± 10.41
P value	≤0.001	≤0.001	≤0.001	0.06
Employment				
Unemployed	69.42 ±14.44	61.27 ±16.63	70.98 ±11.24	68.28 ±10.63
Employed	75.78 ±12.32	68.06 ±15.42	74.15 ±10.53	70.08 ±10.68
P value	≤0.001	≤0.001	≤0.001	≤0.05
SES				
Upper middle	77.06±9.78	71.18 ±14.0	75.0 ±9.12	73.50 ±9.21
Upper lower	72.79 ±13.61	64.61 ±16.14	72.87 ±10.81	69.50 ±10.62
Lower middle	67.95 ±15.27	60.62 ±17.80	69.24± 12.0	66.14 ±10.68
P value	≤0.01	≤0.01	≤0.01	≤0.01
Alcohol				
No	70.65 ±14.31	62.10 ±16.59	71.72 ±11.43	68.48± 10.87
Yes	76.95 ±11.35	70.99 ±14.06	74.31 ±9.44	70.94± 9.79
P value	≤0.001	≤0.001	≤0.01	≤0.01
Smoking				
Yes	72.022±13.92	63.74±16,61	72.19±11.09	69.16±10.66
No	72.611±14.36	67.75±14.58	73.40±10.72	67.75±10.85
P value	0.767	0.08	0.444	0.358
BMI				
Normal	74.35 ±13.10	67.09 ±15.82	72.67 ±11.85	70.23 ±10.83
Underweight	74.64 ±13.39	62.23 ±14.31	70.94 ±14.36	69.52 ±11.48
Overweight	72.27 ±14.00	64.87 ±16.01	72.76 ±10.81	69.50 ±10.57
Obese	68.36 ±14.35	59.14 ±16.01	71.25 ±10.29	66.74 ±10.58
P value	≤0.001	≤0.001	0.515	≤0.02
Blood pressure				
Normal	75.24 ±12.74	65.67 ±17.07	73.01 ±11.96	70.12 ±11.18
Elevated	71.94 ±14.42	63.72 ±16.14	72.74 ±11.16	70.17 ±10.98
Hypertension Stage I	66.56 ±13.59	60.39 ±15.38	70.27 ±9.17	65.69 ±8.81
Hypertension Stage II	65.80±15.89	63.36±16.35	73.27±9.98	69.44±10.89
P value	≤0.001	≤0.01	0.08	≤0.001
Stress				
Low	78.38±10.02	72.02±13.83	75.43±9.45	73.05±9.03
Moderate	65.83±13.84	56.34±13.75	68.80±11.82	64.88±10.47
High	50.14±16.49	34.85±16.17	69.21±8.71	58.214±11.56
P value	≤0.001	≤0.001	≤0.001	≤0.001
Anxiety				
Minimal	74.27±12.55	66.40±15.60	72.92±10.79	69.97±10.56
Mild	64.11±13.63	56.19±13.55	70.37±11.26	66.16±9.39
Moderate	51.40±17.42	39.60±18.16	63.40±13.80	55.53±9.71
Severe	43.66±11.18	36.50±13.86	67.83±12.04	61.66±7.47
P value	≤0.001	≤0.001	≤0.01	≤0.001
Depression				
Minimal	72.81±13.47	65.37±15.47	72.77±10.78	69.71±10.13
Mild	63.33±12.47	44.95±13.00	64.00±12.17	58.66±11.52
Moderate	42.12±10.94	23.50±11.46	61.00±14.02	47.75±11.98
P value	≤0.001	≤0.001	≤0.001	≤0.001

In terms of physical health, participants with normal BMI (physical: 74.35 ± 13.10 vs. 68.36 ± 14.35; psychological: 67.09 ± 15.82 vs 59.14 ± 16.01; environment: 70.23 ± 10.83 vs. 66.74 ± 10.58) and normal blood pressure had significantly higher scores (physical: 75.24 ± 12.74 vs. 65.80±15.89; psychological: 65.67 ± 17.07 vs. 63.36 ± 16.35; environment: 70.12 ± 11.18 vs. 69.44 ± 10.89) of QoL except in the social relation domain compared to participants with higher BMI and blood pressure. With regard to mental health, increase in stress, anxiety, and depression, there was a significant decrease in average scores of QoL across all four domains.

Table 4 depicts the correlation of various predictors with physical health, social relations, psychological domain, environmental domain, and overall means score of WHOQOL-BREF. It was found that the variables, which included an increase in age, female participants, higher BMI, and blood pressure, along with the increased levels of stress, anxiety, and depression, had a significant negative correlation with all four domains of QoL, whereas higher socioeconomic status and employment had a significant positive correlation with QoL. 

**Table 4:** Correlation of various predictors affecting QoL **p-value ≤ 0.001 and *p-value ≤0.05 are considered significant. SES: socioeconomic status, BMI: body mass index, SBP: systolic blood pressure, DBP: diastolic blood pressure, QoL: quality of life.

Variables	Domains of QoL				
	Physical (r)	Social (r)	Psychological (r)	Environment (r)	Total QoL
Age	-0.334**	-0.115**	-0.096*	-0.088*	-0.203**
Gender	-0.303**	-0.146**	-0.235**	-0.075	-0.228**
SES	0.143**	0.123**	0.077	0.126**	0.129**
Employment	0.225**	0.141**	0.203**	0.083*	0.157**
Alcohol consumption	0.189**	0.089*	0.225**	0.096*	0.183**
Smoking status	0.012	0.031	0.070	-0.038	0.017
BMI	-0.207**	-0.041	-0.205**	-0.143**	-0.198**
SBP	-0.199**	-0.034	-0.076	-0.096*	-0.137**
DBP	-0.236**	-0.038	-0.076	-0.097*	-0.151**
Stress	-0.557**	-0.351**	-0.573**	-0.449**	-0.580**
Anxiety	-0.505**	-0.216**	-0.442**	-0.304**	-0.460**
Depression	-0.519**	-0.346**	-0.574**	-0.457**	-0.573**

Table 5 represents the potential influence of factors on QoL domains of WHOQOL-BREF. The multiple linear regression analysis revealed that all domains of QoL and overall scores were independently affected by stress (≤0.001) and depression (≤0.001). Participants detected with higher levels of stress and depression reported a significant decrease in their scores. Increased level of anxiety (≤0.05) independently affects all the domains and overall score except social and environmental domains. BMI (≤0.05) was associated with the psychological domain and overall score, while age affected the physical, social, and overall scores, whereas variables like higher socioeconomic status (≤0.05), employment (≤0.01), and alcohol consumption (≤0.05) reported significantly better scores for physical, social, psychological, and overall scores of QoL. 

**Table 5:** Multilinear regression analysis of QoL with various social and health predictors p-value ≤ 0.001 or ≤0.05 is considered significant. SES: socioeconomic status, BMI: body mass index, SBP: systolic blood pressure, DBP: diastolic blood pressure, QoL: quality of life.

Predictors	Domain of QoL									
	Physical		Psychological		Social		Environment		Total QoL	
	Beta	p	Beta	p	Beta	p	Beta	p	Beta	p
Age	-0.265	≤0.001	0.013	0.769	-0.138	≤0.01	-0.030	0.536	-0.128	≤0.01
Gender	-0.002	0.959	-0.030	0.530	-0.027	0.648	0.112	0.041	0.052	0.272
Employment	0.098	≤0.01	0.027	0.525	0.088	0.090	0.020	0.679	0.060	0.154
SES	0.071	≤0.05	0.013	0.691	0.069	0.07	0.078	≤0.05	0.060	0.060
Smoking	-0.053	0.139	-0.014	0.719	-0.006	0.893	-0.054	0.202	-0.044	0.233
Alcohol	0.075	0.09	0.112	≤0.01	0.005	0.933	0.71	0.178	0.088	≤0.05
BMI	-0.039	0.225	-0.115	≤0.001	0.040	0.330	-0.055	0.178	-0.068	≤0.05
SBP	-0.025	0.590	-0.070	0.149	-0.018	0.757	-0.053	0.156	-0.053	0.267
DBP	-0.010	0.828	0.041	0.391	0.048	0.410	0.003	0.333	-0.016	0.731
Stress	-0.275	≤0.001	-0.288	≤0.001	-0.207	≤0.001	-0.242	≤0.001	-0.295	≤0.001
Anxiety	-0.156	≤0.001	-0.092	≤0.05	0.030	0.568	-0.018	0.704	-0.092	≤0.05
Depression	-0.215	≤0.001	-0.317	≤0.001	-0.223	≤0.001	-0.309	≤0.001	-0.316	≤0.001
R^2^	0.0496		0.436		0.175		0.285		0.462	

## Discussion

The present study assessed QoL and its associated biopsychosocial determinants among the Yadav community of Delhi, India. Among the four domains of QoL, participants in the social domain had the highest proportion of good QoL, followed by the environmental domain. These results reflected a sense of solidarity, good social support from family and friends, and positive feelings in personal relationships among individuals of this community [2,21]. On the contrary, the psychological domain had the lowest proportion of good QoL, indicating more negative feelings about life and poor self-esteem. This result is similar to findings from other studies [3,5,22].

Interesting results were found in the present study regarding varied domain scores according to sociodemographic characteristics. A significant decrease in QoL was observed with an increase in age across physical and social domains, a finding similar to a study conducted in Vietnam [23]. With the increase in age, adults become more dependent on medication or treatments and are less satisfied with their physical health and social relationships [24]. Moreover, there is a need among adults to spread awareness regarding conditions associated with aging as a positive process and old age as a stage of life in which health, well-being, and QoL can be enhanced by focusing on their current health [25]. Similarly, employed participants had significantly higher QoL compared to unemployed participants, in line with findings from another study [26]. These individuals develop confidence and have better self-esteem due to financial independence. Also, a significant decrease in the mean scores of socioeconomic status (SES) from middle SES to lower SES was observed across all domains. Similar findings were reported in other studies, with higher-income individuals associated with good QoL, particularly in relation to health concerns. People with higher socioeconomic status reported better QoL scores [27].

The present study indicated that women have a lower QoL than men. In fact, many studies have reported lower QoL among females, consistent with studies conducted among the Iranian general population that reported poorer QOL for women than men [28]. The authors argued that these differences might be due to marriage at an early age, sociodemographic, and socioeconomic differences. The literature suggests that females have faced unique challenges and experiences that negatively affect their overall well-being post-pandemic [24,29].

In the context of lifestyle variables, alcohol consumption was found to have a statistically significant and positive correlation across all domains of QoL. Similar findings were reported in previous studies; moderate alcohol drinking was found to be positively associated with QoL because of the social factors connected with alcohol consumption. It was seen as one of the few pleasurable activities that included enjoyment, relaxation, and stress relief [30].

Additionally, there were significant correlations related to the overall mean score and general health between the four domains of QoL. Individuals with higher BMI have significantly lower mean scores than individuals with normal BMI across all domains. The literature suggests that a sedentary lifestyle, unhealthy eating habits, excessive behavioral stress, anxiety, and depression have been identified as risk factors for obesity during the COVID-19 pandemic that can impact the QoL among individuals [3,5,8,9]. Moreover, this study found that individuals with hypertension have significantly lower mean scores than normotensive individuals across all domains. Our findings are consistent with previous studies [6,7,31].

Many studies also support the importance of more public education on hypertension prevention, early diagnosis, and effective treatment of chronic conditions to preserve QoL in community-based research to decrease potential adverse consequences [7,9,19,23].

Poor mental health condition is a burden on participants' QoL. The findings of the present study show that the existence of common mental disorders (stress, anxiety, and depression) affects QoL and is also a predictor of reduced QoL; it significantly decreases across all the domains. Several studies on this subject found that the presence of depressive or anxious symptoms has a negative impact on QoL [4,5]. Therefore, it is crucial to identify and address these factors to promote a better QoL among people. By taking steps to improve the QoL, we can help individuals lead a happier, healthier, and more fulfilling life.

Strength and limitations

To the best of our knowledge, this study is the first community-based research conducted in Delhi, India, that assessed all the domains of QoL using the World Health Organization Quality of Life: Brief (WHOQOL-BREF) and its associated biopsychosocial determinants. Additionally, the study assessed the QoL of participants from a holistic perspective. Though this study holds significance in today's scenario, it has a few limitations. Firstly, the study's cross-sectional nature limits the generalizability outside this community and demographic area. It also limits the ability to establish a cause-and-effect relationship between the variables. Furthermore, the data have a higher proportion of female participants.

## Conclusions

Our study found that physical and mental health adversities negatively impact QoL, with participants exhibiting poor QoL in the psychological domain and good QoL in the social domain. Consequently, these adversities affect an individual's overall well-being, leading to decreased productivity, work-life balance, and happiness. The present study suggests that understanding the QoL of individuals with mental disorders, hypertension, and obesity is crucial in assisting policymakers and healthcare providers to develop targeted interventions and policies to promote health and well-being for all. Culture-specific intervention programs should aim to improve QoL by understanding the health needs and risks of different communities in Delhi. Following the outbreak of the coronavirus pandemic, there is an urgent need to increase awareness regarding healthy lifestyles and physical activity (especially leisure time) through community-specific, behavioral intervention programs, considering the diverse sociocultural practices in the country. To some extent, the proposed study has spread awareness of their physical and mental well-being among the participants. Therefore, it is important for individuals to focus on improving their quality of life, as it can significantly impact their overall happiness and success in life.
